# Is Ross Syndrome a New Type of Synucleinopathy? A Brief Research Report

**DOI:** 10.3389/fnins.2020.00635

**Published:** 2020-07-08

**Authors:** Mingming Ma, Jing Yao, Yongkang Chen, Han Liu, Danhao Xia, Haiyan Tian, Xinxin Wang, Erxi Wu, Xuejing Wang, Xuebing Ding

**Affiliations:** ^1^Department of Neurology, Affiliated People’s Hospital of Zhengzhou University, Henan Provincial People’s Hospital, Zhengzhou, China; ^2^Department of Neurology, The First Affiliated Hospital of Zhengzhou University, Zhengzhou, China; ^3^Institute of Parkinson and Movement Disorder, Zhengzhou University, Zhengzhou, China; ^4^Department of Neurosurgery, Neuroscience Institute, Baylor Scott & White Health, Temple, TX, United States; ^5^Health Science Center, Colleges of Medicine and Pharmacy, Texas A&M University, College Station, TX, United States; ^6^Department of Oncology, Dell Medical School, LIVESTRONG Cancer Institutes, The University of Texas at Austin, Austin, TX, United States

**Keywords:** peripheral autonomic system disorder, pure autonomic failure, α-synucleinopathy, neuropathology, autonomic dysfunction

## Abstract

Ross syndrome (RS) is a rare peripheral autonomic system disorder characterized by tonic pupil, hyporeflexia, and segmental anhidrosis. Neuropathological studies show that RS results from the selective cholinergic nerve degeneration. However, the cause and underlying mechanisms are largely unknown. Here, we show α-synuclein accumulation in the autonomic nerve terminals in the lesser curvature of stomach of patients with RS. In addition, immunohistochemical findings demonstrate that a dominant degeneration of cholinergic fibers is exhibited in patients with RS, while main degeneration of adrenergic fibers is demonstrated in patients with pure autonomic failure in their gastrointestinal and urinary system. Our study suggests that RS belongs to α-synucleinopathies. Moreover, our findings indicate that adrenergic nerves and cholinergic nerves are not equally damaged in different types of pure autonomic dysfunctions.

## Introduction

Ross syndrome (RS) is a rare disorder characterized by segmental anhidrosis, tonic pupil, and hyporeflexia first reported by Ross (1958). Sommer et al. (2002) then found a selective loss of cholinergic sudomotor fibers in Ross patients. Interestingly, Nolano et al. (2006) discovered that impairment of heat production and dissipation in Ross patients are associated with cutaneous sensory and autonomic innervation.

Given that the autonomic nervous system features in many neurological disorders, it plays an essential role in regulating physiological homeostasis, for example, in α-synucleinopathies. α-Synucleinopathies are a group of neurodegenerative diseases characterized by the abnormal accumulation of α-synuclein (α-Syn) aggregates in neurons, glial cells, or nerve fibers, including Parkinson’s disease (PD), Lewy body dementia (LBD), and multiple system atrophy (MSA). Pure autonomic failure (PAF) is also suggested to be an α-synucleinopathy, and pathologic findings show that lesions in the peripheral autonomic nervous system of PAF are associated with α-Syn-positive deposits (Thaisetthawatkul, 2016).

In this study, we investigated neuropathological changes or innervation of skin, stomach, and bladder from patients with RS, PAF, PD, or MSA. We found that α-Syn aggregates deposit in autonomic nerve terminals of skin and lesser curvature of stomach in patients with RS. Our findings provide evidence that RS belongs to α-synucleinopathies, which has not been recognized before. Additionally, immunohistochemical studies showed that there is a dominant loss of cholinergic fibers in patients with RS, while the mainly damaged fibers are adrenergic in patients with PAF.

## Materials and Methods

### Subjects

Twenty-three patients (11 men, 12 women; 47–66 years of age) with RS (2 patients), PAF (6 patients), PD (10 patients), or MSA (5 patients) referred to our Neurology Clinic were recruited according to clinical features and consensus diagnostic criteria (Ross, 1958; Gilman et al., 2008; Kalia and Lang, 2015; Thaisetthawatkul, 2016). The normal controls were randomly selected from the gastroscopy clinic patients with superficial gastritis (six subjects) or the cystoscopy clinic patients with chronic cystitis (six subjects); all of them were free of neurological symptoms. Demographic data and autonomic symptoms of the tested patients are summarized in Table 1.

**TABLE 1:** Clinical and demographic data.

**Diagnosis**	**Age, y**	**Sex**	**Disease duration, y**	**Autonomic dysfunction**				
				**SL**	**OH**	**UI**	**IS**	**GP**
RS	50	F	10	+	−	+	−	+
RS	47	M	4	+	−	−	−	+
PAF	49	M	5	+	+	−	+	+
PAF	54	M	1	−	+	+	−	−
PAF	61	F	1	−	+	+	−	−
PAF	58	M	4	−	+	−	−	−
PAF	63	F	6	−	+	+	−	−
PAF	51	M	3	−	+	−	+	−
PD	54	F	10	−	−	−	−	−
PD	58	F	3	−	−	−	−	−
PD	61	M	6	−	−	+	+	−
PD	65	F	4	−	−	+	+	−
PD	54	F	10	−	−	−	+	−
PD	56	F	1	−	−	−	+	−
PD	54	F	3	−	−	+	−	−
PD	63	M	1	−	+	+	−	−
PD	54	M	1	−	−	+	+	−
PD	64	F	8	−	−	+	−	−
MSA	66	F	3	−	−	+	+	−
MSA	54	M	2	−	−	+	+	−
MSA	59	M	2	−	−	+	+	−
MSA	50	M	2	−	−	−	+	−
MSA	57	F	8	−	−	+	+	−

*+, abnormal finding; −, normal finding. F, female; GP, gastrointestinal paresis; IS, impotence and sexual dysfunction; M, male; MSA, multiple system atrophy; OH, orthostatic hypotension; PAF, pure autonomic failure; PD, Parkinson’s disease; RS, Ross syndrome; SL, sweat loss; UI, urinary incontinence; y, years.*

### Sweating Function

#### Thermoregulatory Sweat Test (TST)

TST was performed as previously described (Nolano et al., 2006). The subject’s face was covered with a 2% alcoholic solution of iodine and with rice starch powder except for eyes in a prescriptive room. The color of the rice starch powder changes to black while sweating. Digital pictures were taken using a Canon camera (EOS 200D II, Canon).

#### Digital Infrared Thermal Imaging (DITI)

DITI was performed according to a previous publication (Kontos et al., 2011). The subjects removed clothing and sat quietly in a prescriptive room. Colored pictures were taken using the DITI system. Black, purple, blue, green, yellow, brown, red, and grayish white in the pictures represented temperatures from low to high.

### Skin, Gastric, and Bladder Biopsies

Biopsies were conducted by experienced endoscopists or urologists in a procedure room. Skin samples were obtained from the left (hyperhidrotic site) and right (anhidrotic site) inner aspect of the upper arms in patients with RS. Gastric biopsy samples from patients were acquired from the lesser curvature and antrum regions during percutaneous endoscopic gastroscopy, and the samples from normal controls were randomly selected from gastric biopsies in gastritis patients who attended the gastroscopy clinic. Bladder tissues from patients were obtained during cystoscopy, and the tissues from normal controls were randomly selected from bladder biopsies in cystitis patients who attended the cystoscopy clinic. All biopsies were immediately fixed in 30% sucrose solution containing 4% paraformaldehyde and embedded in paraffin blocks. Immunohistochemical and immunofluorescence analyses were performed as described before (Donadio et al., 2013). For immunohistochemical analysis, endogenous tissue peroxidases were inhibited by incubating the slides in 3% hydrogen peroxide solution. Slides were then blocked with normal goat serum for 20 min and then incubated with primary antibodies: Ser129phospho-α-Syn (pα-Syn) (1:600, mouse, Millipore, MABN826), α-Syn filament (1:500, rabbit, Abcam, ab209538), and protein gene product 9.5 (PGP 9.5) (1:300, rabbit, Abcam, ab108986). Bound antibodies were detected using a Streptavidin-Peroxidase kit (Bioss and Proteintech, China) and visualized using 3-3′-diaminobenzidine (DAB; Neobioscience). Slides were counterstained with hematoxylin. For immunofluorescence analysis, slides were blocked with 5% BSA for 30 min at room temperature and then incubated with combinations of primary antibodies: Ser129phospho-α-Syn (pα-Syn) (1:800, mouse, Millipore, MABN826), PGP 9.5 (1:300, rabbit, Abcam, ab108986), vasoactive intestinal peptide (VIP) (1:300, mouse, Abcam, ab30680), Tyrosine Hydroxylase (TH) (1:600, mouse, Santa Cruz Biotechnology, sc-25269), and anti-Vesicular Acetylcholine Transporter (VAChT) (1:300, goat, Millipore, ABN100). Cell nuclei were stained using Hoechst 33258 (1:1000, Solarbio, C0021). Digital images were captured using an Olympus IX51 microscope mounted with a DP71 Olympus digital camera. Photoshop CS6 (Adobe Systems) was used to assemble montages. Data from immunohistochemistry and immunofluorescence were quantified using ImageJ Software.

### Statistical Analysis

Statistical analysis of behavioral data was performed using SPSS 21.0 (IBM, Armonk, NY, United States). Student’s *t*-test was employed for comparison between two groups, while one-way ANOVA was used for three groups when the data were distributed normally (*P <* 0.05 by Shapiro–Wilk test). Otherwise, the analysis of variance corrected for multiple comparisons was utilized. *P* < 0.05 was considered statistically significant.

## Results

### Clinical Characteristics and Autonomic Symptoms

**FIGURE 1:**
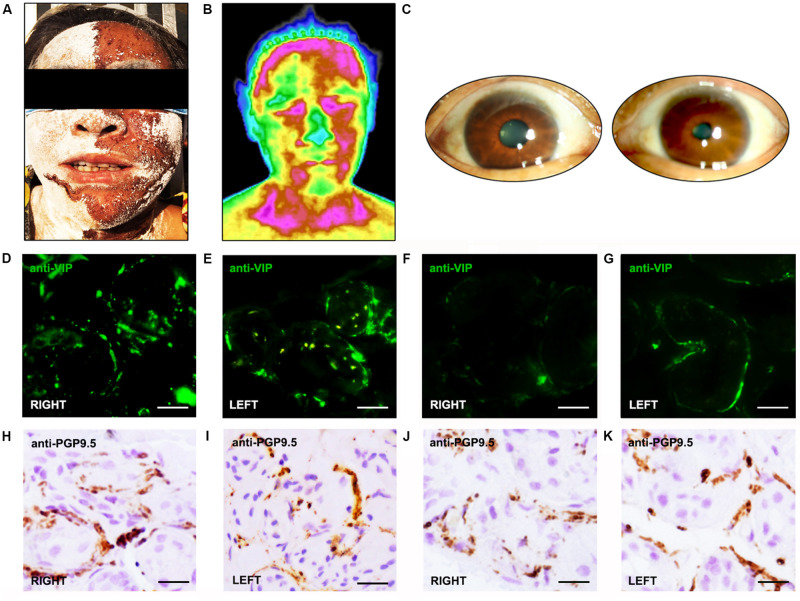
Morphologic and functional features of patients and normal controls. **(A)** Thermoregulatory sweat test (iodine-starch method). Hypohidrosis is seen mainly on the right side of the face. **(B)** Digital infrared thermal imaging shows a lower temperature in the left side of the face than the right. **(C)** Pupillary light reflex appears sluggish in the right eye, but normal in the left eye. **(D–G)** VIP immunoreactive fibers appeared more frequently in the skin of normal controls **(D,E)** than in Ross patients **(F,G)**. In Ross patients, rare VIP immunoreactive fibers were found around sweat glands in anhidrotic skin **(F)**, while VIP-ir fibers appeared more frequently in hyperhidrotic skin **(G)**. **(H–K)**. The PGP 9.5-ir fibers appeared more frequently in the skin of normal controls **(H,I)** than in Ross patients **(J,K)**. In Ross patients, the PGP 9.5-ir fibers were severely reduced in anhidrotic skin **(J)** and slightly reduced in hyperhidrotic skin **(K)**.

Twenty-three subjects with RS, PAF, PD, or MSA participated in the study. Among these subjects, two patients with RS presented with unilateral anhidrosis and bilateral tonic pupils (Figure 1C), their blood tests and physical examination were normal, but neurological examination revealed diffuse bilateral hyporeflexia in all the four limbs. Furthermore, TST confirmed segmental anhidrosis involved in the body surface of Ross patients (Figure 1A), and DITI revealed that the mean skin temperature of the anhidrosis area was higher than that of the hyperhidrosis area (Figure 1B). Additionally, various symptoms of autonomic dysfunction identified in patients participated are shown in Table 1. Both patients with RS displayed segmental sweat loss and gastrointestinal paresis. All PAF patients had orthostatic hypotension.

### Pathological α-Syn Deposits in Skin and Gastric Nerve Terminals of Patients With RS

**FIGURE 2:**
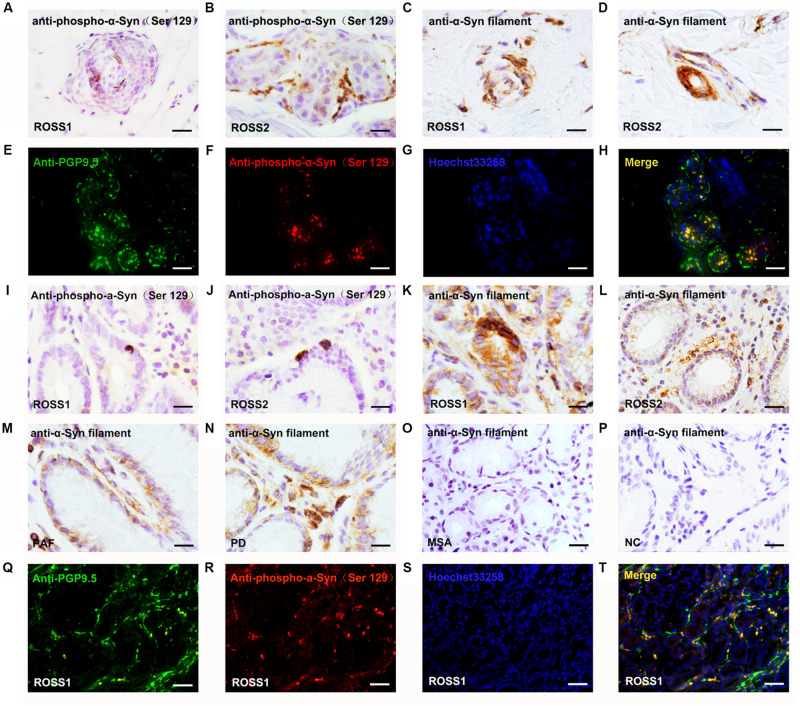
Pathological α-Syn immunoreactivity in patients with Ross syndrome, PAF PD, MSA, and normal controls. **(A–D,I–P)** Some of fibers were positive for pα-Syn inclusions **(A,B,I,J)** or α-Syn filament **(C,D,K,L)** in anhidrotic skin **(A–D)** and lesser curvature of stomach **(I–L)**. **(M–P)** α-Syn filament was present in lesser curvature of stomach in PAF **(M)** and PD **(N)**, but not in MSA **(O)** and normal controls **(P)**. **(E–H,Q–T)** Double immunofluorescence analysis showed that pα-Syn colocalized with panaxonal marker PGP 9.5 in the skin **(E–H)** and lesser curvature of stomach **(Q–T)** from the patients with RS. Bar = 100 μm.

Immunohistochemical analysis revealed that VIP-immunoreactive (VIP-ir) fibers around sweat glands were severely decreased in the skin of Ross patients than in the skin of normal controls (Figures 1D–G). Moreover, rare VIP-ir fibers were found in anhidrotic skin of Ross patients (Figure 1F), while they were moderately decreased in hyperhidrotic skin (Figure 1G). In addition, we observed a slightly increased loss of PGP 9.5-ir fibers in the skin of Ross patients than in normal controls (Figures 1H–K). Moreover, PGP 9.5-ir fibers were severely reduced in Ross patients’ anhidrotic skin (Figure 1J) than in hyperhidrotic skin (Figure 1K). Furthermore, phospho-α-Syn-ir (pα-Syn-ir) inclusions and α-Syn filament were found in nerve terminals in dermis and subcutaneous tissues of Ross patients (Figures 2A–D). To determine whether pathological α-Syn accumulates in nerve terminals of gastrointestinal tract and bladder, we performed immunohistochemistry on gastric and bladder samples using anti-pα-Syn (Ser129pα-Syn, mouse, Millipore) and anti-α-Syn filament (rabbit, Abcam) antibodies. We found that pathological α-Syn deposited in the lesser curvature of stomach in both of the Ross patients (100%), 6 out of 10 PD cases biopsied (60.0%), 2 of 6 PAF cases (33.3%), but none from MSA patients or normal controls (Figures 2I–P). Moreover, double-immunofluorescent staining showed that pα-Syn was deposited within nerve terminals in the skin (Figures 2E–H) and surrounding gastric glands of RS patients (Figures 2Q–T). No α-Syn-positive inclusions were detected in bladder tissues of RS patients (data not shown).

### Selective Loss of Cholinergic Innervation in RS Patients and Adrenergic Innervation in PAF Patients in Gastrointestinal and Urinary System

**FIGURE 3:**
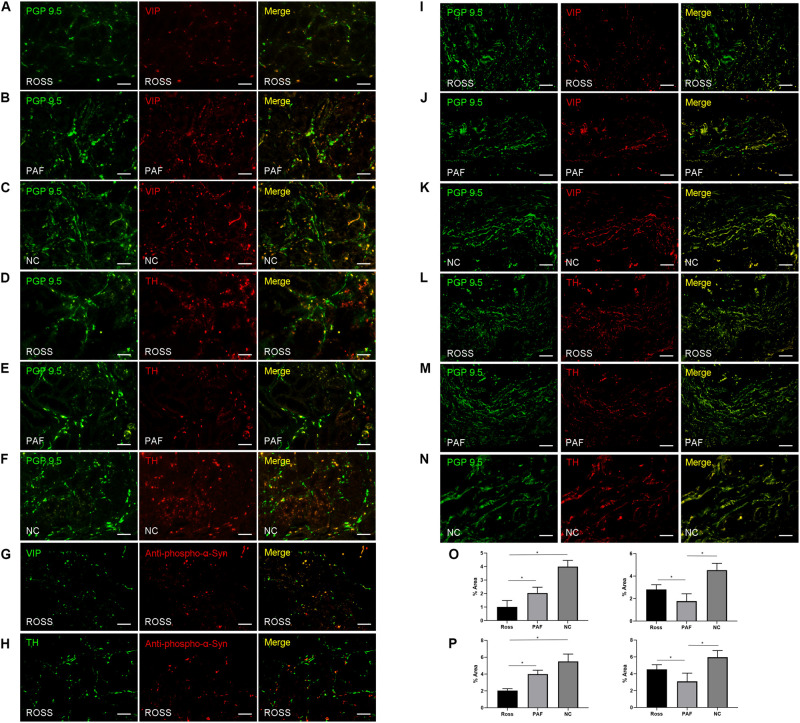
Histopathological features in patients with Ross syndrome, PAF, and normal controls. **(A–F)** Double immunofluorescence analysis of lesser curvature of stomach: Nerve fibers are marked in green using PGP 9.5; cholinergic fibers and adrenergic fibers are stained in red by anti-VIP and anti-TH antibodies. VIP-ir fibers appear poorly represented in RS **(A)**, while they were evident in PAF **(B)** and normal controls **(C)**. TH-ir fibers were present in Ross patients **(D)** and normal controls **(F)**, while they were reduced in PAF **(E)**. **(G,H)** Double immunofluorescence analysis of lesser curvature of stomach; VIP-ir pα-Syn **(G)** appeared more frequently than TH-ir pα-Syn **(H)**. **(I–N)** Double immunofluorescence study of urinary bladder innervation. VIP-ir fibers appear poorly represented in RS **(I)**, while they were evident in PAF **(J)** and normal controls **(K)**. TH-ir fibers were present in Ross patients **(L)** and normal controls **(N)**, while they were reduced in PAF **(M)**. **(O,P)** Quantification of VIP-ir fibers and TH-ir fibers in the lesser curvature of stomach **(O)** and bladder **(P)**, respectively. Bar = 100 μm. *P < 0.05.

PGP 9.5-ir nerve fibers in stomach were significantly decreased in patients with RS and PAF, compared with normal controls (Figures 1H–K). Double immunofluorescence analysis showed that VIP-ir nerve fibers were profoundly decreased in lesser curvature of stomach from RS patients (Figures 3A–C,O), while TH-ir nerve fibers were decreased in lesser curvature of stomach from PAF (Figures 3D–F,O). These findings indicate that there is a preferential loss of cholinergic innervation in RS patients, while predominant loss of adrenergic innervation in PAF patients in the gastrointestinal tract. Furthermore, VIP-ir pα-Syn appeared more frequently than TH-ir pα-Syn in the lesser curvature of stomach (Figures 3G,H). In addition, immunofluorescence analytical result of bladder biopsy showed selective loss of cholinergic innervation in RS patients (Figures 3I–K) and adrenergic innervation in PAF patients in bladder (Figures 3K–N,P).

## Discussion

Bergmann et al. (1998) reported that RS is a rare peripheral autonomic dysfunction characterized by selective loss of cholinergic fibers. To date, the underlying disease mechanism remains unclear (Weller et al., 1992; Perretti et al., 2003; Sawhney et al., 2004; Nagane and Utsugisawa, 2008; Vasudevan et al., 2010). α-Synucleinopathies share the pathological hallmark of α-Syn insoluble inclusions in neurons, glial cells, or nerve fibers (Kiely et al., 2013) and often present with prominent autonomic symptoms. Our findings show that pathological α-Syn deposited in the autonomic nerve terminals of samples from patients with RS. It provides preliminary evidence that RS may belong to α-synucleinopathies. We surmise that selective loss of cholinergic fibers may be associated with accumulations of pathological α-Syn in the nerve terminals. These findings may shed new light on the correlation between disorders of peripheral autonomic nervous system and α-synucleinopathy, which warrants further investigation.

Furthermore, immunofluorescence analysis revealed that gastric and urinary bladder innervation was decreased in patients with RS and PAF. Moreover, we found selective loss of cholinergic innervation in patients with RS while adrenergic innervation in patients with PAF. It is suggested that different phenotypes of autonomic failure may be attributed to pathological changes of different types of autonomic nerve fibers. However, this current study is limited by its small sample size and our preliminary findings will certainly need to be confirmed by future work.

## Conclusion

Our study suggests that RS belongs to α-synucleinopathies. Moreover, our findings indicate that adrenergic nerves and cholinergic nerves are not equally damaged in different types of pure autonomic dysfunctions.

## Data Availability Statement

The raw data supporting the conclusions of this article will be made available by the authors, without undue reservation, to any qualified researcher.

## Ethics Statement

The studies involving human participants were reviewed and approved by the Institutional Ethics Committees of the Zhengzhou University. The patients/participants provided their written informed consent to participate in this study. Written informed consent was obtained from the individual(s) for the publication of any potentially identifiable images or data included in this article.

## Author Contributions

MM conceived and designed the experiments. XD and XJW coordinated the whole project. MM, JY, and YC conducted the histological preparations. XD, JY, HT, HL, and DX performed the immunostaining analysis. HL, XXW, and MM performed the TST and DITI. XXW, HT, XD, and YC participated in the final data analysis and interpretation. XJW, EW, and JY did most of the writing with input from other authors. All authors contributed to the article and approved the submitted version.

## Conflict of Interest

The authors declare that the research was conducted in the absence of any commercial or financial relationships that could be construed as a potential conflict of interest.
